# *Mycoplasma pneumoniae* and *Chlamydia pneumoniae* Coinfection with Acute Respiratory Distress Syndrome: A Case Report

**DOI:** 10.3390/diagnostics12010048

**Published:** 2021-12-27

**Authors:** Meng-Ko Tsai, Chao-Hung Lai, Chris Tsai, Guan-Liang Chen

**Affiliations:** 1 Department of Internal Medicine, Division of Allergy, Immunology, and Rheumatology, Taichung Armed Forces Taichung General Hospital, Taichung 41152, Taiwan; raymondpaper@gmail.com; 2 Department of Internal Medicine, Division of Allergy, Immunology, and Rheumatology, Tri-Service General Hospital, National Defense Medical Center, Taipei 11490, Taiwan; 3 Graduate Institute of Medical Sciences, National Defense Medical Center, Taipei 11490, Taiwan; 4 Department of Internal Medicine, Division of Cardiology, Taichung Armed Forces General Hospital, Taichung 41152, Taiwan; lchmed077@gmail.com; 5 Genetics Generation Advancement Corporation, Taipei 11490, Taiwan; pamelalo@gga.asia or; 6 Department of Internal Medicine, Division of Chest Medicine and Respiratory Therapy, Taichung Armed Forces General Hospital, Taichung 41152, Taiwan; 7 Department of Internal Medicine, Division of Chest Medicine and Respiratory Therapy, Tri-Service General Hospital, National Defense Medical Center, Taipei 11490, Taiwan

**Keywords:** *Mycoplasma pneumoniae*, *Chlamydia pneumoniae*, acute respiratory distress syndrome, leucine-rich repeat-containing 16A

## Abstract

Community-acquired pneumonia caused by *Mycoplasma pneumoniae* or *Chlamydia pneumoniae* is usually mild. *Mycoplasma pneumoniae*-related and *C. pneumoniae*-related acute respiratory distress syndromes (ARDSs) are rare. Moreover, to our knowledge, there are no published reports on ARDS caused by *M. pneumoniae* and *C. pneumoniae* coinfection. Here, we report a case of an immunocompetent young woman who was co-infected with *M. pneumoniae* and *C. pneumoniae* and was started on treatment with piperacillin and clarithromycin. Two days later, she developed ARDS. She recovered rapidly following a change of antibiotic treatment to levofloxacin and was discharged on day 12. We conducted exome sequencing followed by alternative filtering to search for candidate ARDS-related genes. We identified an intronic variant of unknown significance within leucine-rich repeat-containing 16A (*LRRC16A*), a gene previously identified as a significant locus for platelet count with a possible role in ARDS. This is a rare case of ARDS in a young adult caused by *M. pneumoniae* and *C. pneumoniae* coinfection. This case suggests that ARDS in young adults may be correlated with variants in *LRRC16A*. This requires confirmation by further case reports.

## 1. Introduction

Acute respiratory distress syndrome (ARDS) is an acute diffuse, inflammatory lung injury associated with increased pulmonary vascular permeability. ARDS is relatively common and is associated with high mortality. Histology shows diffuse alveolar damage that can be induced by either direct lung injury (e.g., pneumonia and aspiration) or indirect lung injury (e.g., sepsis, non-thoracic trauma, and pancreatitis) [1]. Pneumonia and sepsis are the most common causes of ARDS.

Community-acquired pneumonia (CAP) is frequently caused by *Mycoplasma pneumoniae* and *Chlamydia pneumoniae* [2] and is often self-limiting [3,4,5]. Coinfection of *Mycoplasma pneumoniae* and *Chlamydia pneumoniae* is not uncommon, especially in children [6,7,8]. However, cases of *M. pneumoniae*-related and *C. pneumoniae*-related ARDS are rare in adults. A retrospective review of reports of *M. pneumoniae* infection with hypoxemia, published in the English and Japanese literature from 1979 to 2010, found only 52 cases (mostly in adults) [9] and did not identify any obvious risk factors. Another retrospective study included 40 patients diagnosed with *C. pneumoniae* in a medical center in Japan from 1996 to 2001. None of the patients in this case series were admitted to the intensive care unit. Advanced age has been identified as a possible risk factor of *C. pneumoniae*-related ARDS [10].

Our literature review did not identify any cases of ARDS caused by *M. pneumoniae* and *C. pneumoniae* coinfection in adults. Although significant improvement has been made in determining clinical risk factors in patients with ARDS, clinical risk factors alone do not predict which patients develop ARDS. Thus, there may be genetic factors that predispose patients to ARDS. Several studies have shown that variants in genes, such as surfactant protein B gene, angiotensin-converting enzyme gene, and *LRRC16A* may be associated with an increased risk of ARDS [11]. However, most studies on ARDS risk have not included younger adults.

Here, we report a rare case of ARDS caused by *M. pneumoniae* and *C. pneumoniae* coinfection in an immunocompetent young woman. We performed whole-exome sequencing (WES) to try to identify possible genetic susceptibility factors.

## 2. Case Presentation

**Figure 1:**
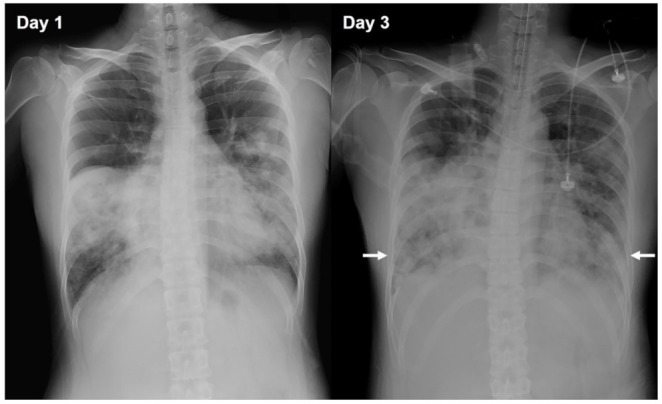
Chest radiographs showing the progression of pneumonia. Chest radiograph on day 1 (**left**) and day 3 (**right**). The radiograph performed on day 3 shows the progression of the pneumonia, with patchy opacification in the lower lobes of the lungs (arrows).

A 31-year-old woman without systemic disease presented to the emergency department with a 10-day history of a productive cough and fever. She did not have a history of susceptibility to respiratory infections. Her daughter had been diagnosed with pneumonia 2 weeks earlier and her niece had a common cold 2 days earlier. On admission, the patient was febrile (38.5 °C) and had bilateral rhonchi in the lower lungs, a white blood cell count of 15,400 × 10^3^ cells/μL, and a platelet count of 90 × 10^3^/μL. On day 1, she was treated with 4.5 g of piperacillin-tazobactam every 8 h and clarithromycin 500 mg every 12 h for bacterial pneumonia. On day 2 she developed progressive dyspnea and was transferred to the intensive care unit (ICU) for intubation. On ICU admission, her ventilator setting was at a pressure control level of 16 cm H_2_O with a positive end-expiratory pressure of 8 cm H_2_O and a PaO_2_:FiO_2_ ratio of 198. Chest radiography revealed rapid development of patchy opacities in the lower lungs (Figure 1). We suspected that she had atypical pneumonia-related ARDS. On day 3, the antibiotic treatment was changed to levofloxacin (750 mg per day) because of suspected clarithromycin resistance. Follow-up blood tests revealed white blood cell and platelet counts of 8400 × 10^3^ cells/μL and 230 × 10^3^/μL, respectively. Assays for atypical pneumonia pathogens revealed a positive cold agglutinin titer (1:64), *Chlamydia* immunoglobulin M (IgM) positivity (1.51), and a high *Mycoplasma* IgM titer (75.01 Bethesda units/mL). Real-time polymerase chain reaction (PCR) assay results for influenza viruses in nasopharyngeal aspirates were negative. The patient’s respiratory condition steadily improved. She was extubated on day 7 and discharged on day 12.

Because of the rarity of *M. pneumoniae*/*C. pneumoniae*-associated ARDS, we discussed the benefits of gene sequencing with our patient, and she provided signed informed consent for further genetic testing. WES did not detect any ARDS-related genetic variants classified as pathogenic or likely pathogenic according to the American College of Medical Genetics and Genomics guidelines. Based on a suggestion by a member of the institution’s genetic laboratory staff, we extracted all the variants identified in the patient’s sample and filtered them according to known ARDS-related genes. Except for six variants, all were classified as benign. The remaining six variants were classified as variants of unknown significance (VUS) and were found in *LRRC16A*, gamma-glutamyl hydrolase, surfactant protein B, transforming growth factor beta receptor 3, thrombospondin 1, and toll-like receptor 1 genes (Table S1). The VUS within *LRRC16A* was an intronic variant, rs1226748546. Five variants (rs1226748546, rs1034051, rs10456324, rs1012899, and rs913455) were found in *LRRC16A* in our patient. However, except for rs1226748546, which was classified as a VUS, the remaining variants were classified as benign (Table S2). During the 1-year follow-up period, the patient did not experience a recurrence of pneumonia or any severe infections.

## 3. Discussion

Since 2000, several genes have been identified that are associated with a risk of ARDS, including surfactant protein B, angiotensin-converting enzyme, nuclear factor erythroid-derived 2-like 2, and serine protease inhibitor (serpin) genes. These genes are listed by the Human Genome Organization (HUGO) as HUGO gene nomenclature committee-approved symbols and are reported annually. We extracted all genetic variants identified in our patient and screened for ARDS-related genes as described by Reilly et al. [12]. We discovered that the VUS in *LRRC16A* (rs1226748546) was an intronic variant.

Platelets are known to make a significant contribution to ARDS of pulmonary origin in critically ill patients by causing endothelial damage. *LRRC16A* encodes the capping protein ARP2/3 and myosin-I linker (CARMIL), which is essential for actin-based cellular processes. Actin-based cellular processes, in turn, are important for megakaryocyte maturation. It has been proposed that the intronic single nucleotide polymorphism (SNP), rs7766874, is in linkage disequilibrium with latent functional variants that alter CARMIL activity, resulting in abnormal megakaryocyte maturation and altered platelet formation [13]. The intronic SNP, rs1226748546, identified in our patient may have exerted a similar effect. In addition, toll-like receptors, which are initiators of innate immune responses that recognize the molecular patterns of invading pathogens—including fungi, bacteria, and viruses—may also contribute to ARDS [14]. All six variants identified in our patient, especially the variant in *LRRC1*6A, may be associated with ARDS; however, this association needs to be confirmed in future studies.

The first-line treatment of CAP comprises a combination of a beta-lactam and a macrolide antibiotic (clarithromycin 500 mg twice daily or azithromycin 500 mg daily) [15]. Our patient showed early treatment failure with the standard regimen. However, after changing the antibiotic to levofloxacin, her symptoms improved rapidly. This may be due to a high rate of resistance to clarithromycin in Asia [9,16]. Inappropriate treatment is a risk factor of fulminant *M. pneumoniae* infection [17].

Mycoplasmas are the smallest prokaryotic pathogens. The pathogenesis of *M. pneumoniae* infection is complex and can be divided into two categories: direct damage and immune damage [18]. CAP caused by *M. pneumoniae* is usually mild; however, life-threatening events may occur in some patients, especially young healthy adults [16]. In fulminant *M. pneumoniae* infection, cell-mediated immune responses may play an immunopathogenic role, exacerbating the lung injury. This may explain why severe *Mycoplasma* infection occurs predominantly in young, previously healthy adults. In addition, animal experiments have shown immune responses to a second infection after *M. pneumoniae* infection, which may explain why *M. pneumoniae* coinfection with *C. pneumoniae* caused ARDS in a young, healthy adult.

Real-time PCR is a valuable tool for diagnosing *M. pneumoniae* and *C. pneumoniae* infection. However, one study that compared serology and PCR testing for diagnosing *M. pneumoniae* infection found that the sensitivity of serological testing for immunoglobulin M (IgM) was higher than that of real-time PCR testing in the first 21 days after symptom onset (76% vs. 48%), but that the specificity of serology was lower than that of PCR testing (63% vs. 98%). No single test is reliably 100% accurate for the identification of *M. pneumoniae* [19]. Similarly, another study on diagnostic testing for C. pneumoniae found that the sensitivity of PCR was lower than that of IgM assays (68% vs. 79–88%), but that the specificity of PCR testing was lower than that of IgM testing (93% vs. 78–86%) [20]. The lack of data on PCR is a limitation of this study. However, serological method may also be reliable for diagnosing *M. pneumoniae* and *C. pneumoniae* infections.

## 4. Conclusions

This rare case of ARDS in an immunocompetent young adult caused by *M. pneumoniae* and *C. pneumoniae* coinfection illustrates that *M. pneumoniae* and *C. pneumoniae* coinfection can cause life-threatening complications, even in an immunocompetent adult. Furthermore, we identified a genetic variant (LRRC16A) in our patient, which may be correlated with ARDS in young adults.

## Data Availability

The data presented in this study are available on request from the corresponding author.

## Supplementary Materials

The following are available online at https://www.mdpi.com/article/10.3390/diagnostics12010048/s1, Table S1: Variants of unknown significance identified in our patient; Table S2: *CARMIL1* (*LRRC16A*) variants identified in our patient.
