# Supplementary material for: Mycoplasma pneumoniae and Chlamydia pneumoniae Coinfection with Acute Respiratory Distress Syndrome: A Case Report
Source: Diagnostics (Basel). 2021 Dec 27;12(1):48. doi: 10.3390/diagnostics12010048 (PMC8775183; doi:10.3390/diagnostics12010048)
Supplement: Supplementary file 1 [file diagnostics-12-00048-s001.zip › Table_S1.pdf]

Table S1. Variants of unknown significance identified in our patient.

Gene	Type	Position	HGVS	Genotype	Allele frequency	ACMG level	SNP
CARMIL1	.	chr6:25426805-25426805	c.249+17G>C	het	4.04279e-06	VUS	rs1226748546
GGH	.	chr8:63948187-63948187	c.224+29dup	het	.	VUS	.
SFTPB	nonsynonymous SNV	chr2:85892764-85892764	c.583G>A	het	0.000766877	VUS	rs184494733
TGFBR3	.	chr1:92185059-92185059	c.1414-38T>A	het	0.00134032	VUS	rs61748118
THBS1	.	chr15:39886402-39886402	c.3365+5G>A	het	0.000270602	VUS	rs185847032
TLR1	nonsynonymous SNV	chr4:38799444-38799444	c.1009C>T	het	0.000127363	VUS	rs200457447

CARMIL, capping protein ARP2/3 and myosin-I linker; SNV, single nucleotide variant; SNP, single nucleotide polymorphism; VUS, variant of unknown significance; HGVS, Human Genome Variation Society; het, heterozygous; ACMG, American College of Medical Genetics.
