# Supplementary material for: Mycoplasma pneumoniae and Chlamydia pneumoniae Coinfection with Acute Respiratory Distress Syndrome: A Case Report
Source: Diagnostics (Basel). 2021 Dec 27;12(1):48. doi: 10.3390/diagnostics12010048 (PMC8775183; doi:10.3390/diagnostics12010048)
Supplement: Supplementary file 1 [file diagnostics-12-00048-s001.zip › Table_S2.pdf]

Table S2. *CARMIL1* (*LRRC16A*) variants identified in our patient.

Sample	Gene	Type	Position	HGVS	Location	SNP	Genotype	Allele Frequency	ACMG Level
WS108044	*CARMIL1*	.	chr6:25492296-25492296	c.1220+44C>T	Intron	rs1034051	hom	0.971119	Benign
WS108044	*CARMIL1*	synonymous SNV	chr6:25600968-25600968	c.3546G>A	Exon	rs10456324	hom	0.434446	Benign
WS108044	*CARMIL1*	nonsynonymous SNV	chr6:25605091-25605091	c.3604G>A	Exon	rs1012899	het	0.768002	Benign
WS108044	*CARMIL1*	.	chr6:25426805-25426805	c.249+17G>C	Intron	rs1226748546	het	4.04279e-06	VUS
WS108044	*CARMIL1*	synonymous SNV	chr6:25420344-25420344	c.141C>G	Exon	rs913455	hom	0.954495	Benign

CARMIL, capping protein ARP2/3 and myosin-I linker; SNV, single nucleotide variant; HGVS, Human Genome Variation Society; SNP, single nucleotide polymorphism; hom, homozygous; het, heterozygous; ACMG, American College of Medical Genetics; VUS, variant of unknown significance.
